# Development of a Computerized Adaptive Test for Problematic Mobile Phone Use

**DOI:** 10.3389/fpsyg.2022.892387

**Published:** 2022-05-31

**Authors:** Xiaorui Liu, Hui Lu, Zhao Zhou, Miao Chao, Tour Liu

**Affiliations:** ^1^Faculty of Psychology, Tianjin Normal University, Tianjin, China; ^2^School of Psychology, Beijing Normal University, Beijing, China; ^3^Key Research Base of Humanities and Social Sciences of the Ministry of Education, Academy of Psychology and Behavior, Tianjin Normal University, Tianjin, China; ^4^Tianjin Social Science Laboratory of Students’ Mental Development and Learning, Tianjin, China

**Keywords:** problematic mobile phone use, computerized adaptive testing, item banking, item response theory, paper-and-pencil test

## Abstract

The great number of mobile phone users in the world has increased in recent years. More time spent on a phone, more negative effects such as problematic mobile phone use. Many researchers have devoted themselves to revise tools to measure problematic mobile phone use better and more precisely. Previous studies have shown that these tools have good reliability and validity, but that most of them have some shortcomings because they were traditional paper-and-pencil tests based on Classical Test Theory (CTT). This study, based on Item Response Theory (IRT) in order to solve these shortcomings, developed Computerized Adaptive Test for problematic mobile phone use (CAT-PMPU) and discussed the performance of CAT-PMPU. Then, we used real data to simulate CAT, and the measurement accuracy and reliability between a paper-and-pencil test and CAT-PMPU were compared under the same test length. The results showed that CAT-PMPU was better than the paper-and-pencil test in all aspects, and that it can reduce the number of items and improve measurement efficiency effectively. In conclusion, the CAT-PMPU was developed in this study has good reliability, and it provided novel technical support for the measurement of problematic mobile phone use. It had a good application prospect.

## Introduction

There were 5.29 billion mobile phone users worldwide in 2021, an increase of 100 million compared with 2020 according to Digital Reports (2021). Peking University Post-95 Mobile Phone Use Psychological and Behavioral White Paper reported that individuals spend more than 8.33 h per day on their phone (Wang et al., 2019). Mobile phone use brought convenience to people’s social communication (Gul et al., 2018) and leisurely experience (Lee et al., 2014; Ra et al., 2018). However excessive mobile phone use also led to many negative consequences such as sleep disorders (Acharya et al., 2013), distress (Chóliz, 2010), and mental health problems (Gezgin and Çakir, 2016; Gezgin, 2017). At present, there are more and more research studies on excessive mobile phone use and mental health.

Different concepts about excessive mobile phone use were put forward in different studies. Toda et al. (2008) defined it as “mobile phone dependence,” a more common term was mobile phone addiction (Chóliz, 2010), and another definition was smartphone addiction (Su et al., 2014). Shotton (1989) and Griffiths (1998) believed that excessive use of technology should be characterized as problematic use rather than addiction. Later, researchers also argued whether mobile phone addiction should be fully considered as an addictive behavior (Bianchi and Phillips, 2005; Tossell et al., 2015). Recently, problematic mobile phone use (PMPU) was widely used in studies (Hao and Jin, 2020; Li et al., 2020). PMPU refers to an uncontrolled and excessive mobile phone use that adversely affects individual’s daily life (Billieux, 2012). PMPU was defined from both psychological and physiological aspects as inappropriate use of mobile phone that would affect personal life and study, and show withdrawal symptoms in the Chinese culture (Xiong et al., 2012).

A review on the prevalence of PMPU in China showed that the prevalence of PMPU ranged from 14 to 31.2% among children and from 27.6 to 29.8% among young people, indicating higher risk of PMPU among young people in China (Lai et al., 2020). Many risk factors of PMPU were revealed, such as residence, depressive symptoms, social anxiety, loneliness, academic pressure, professional interests, family conflicts, neuroticism, openness, and solitude behaviors (Liu et al., 2020; Lu et al., 2021, 2022; Wang and Shi, 2021). Cognitive and executive functions (e.g., attention function and response inhibition function) were proved to be affected if individuals had PMPU (Zhou et al., 2015).

A variety of instruments with good reliability and validity has been developed to measure PMPU. Xiong et al. (2012) developed the Mobile Phone Addiction Tendency Scale (MPATS) to measure improper use of mobile phones. Su et al. (2014) developed the Smartphone Addiction Scale for College Students (SAS-C) to measure the psychological and behavioral performance caused by smartphone abuse. Chen et al. (2017) developed the Smartphone Addiction Scale for Chinese Adults (SAS-CA), which was adapted from college students to adults for assessing PMPU. Basu et al. (2021) developed the Problematic Use of Smartphone Questionnaire (PSUQ) for adults in India. Billieux et al. (2008) developed the Problematic Mobile Phone Use Questionnaire (PMPUQ); subsequently, the short version of PMPUQ showed measurement invariance of various language versions (i.e., French, German, and Hungarian), and could be used in cross-culture research (Olatz et al., 2018).

Most of these PMPU instruments were developed based on symptomology. In other words, each dimension of these PMPU instruments described a typical symptom of PMPU in individuals. MPATS contained four dimensions, withdrawal symptoms, salience, social, comfort, and mood changes. The SAS-C was developed based on the diagnostic criteria of internet addiction proposed by Young (1998). It contained four symptoms, withdrawal behavior, salience behavior, social comfort, and negative effects. The Chinese version of SAS-CA also had four subscales, withdrawal, salience, social impairment, and somatic discomfort. The Smartphone Addiction Proneness Scale (SAPS) and Smartphone Addiction Inventory (SPAI) were developed based on the existing internet addiction literature (Kim et al., 2014; Lin et al., 2014). The SAPS had four subscales, including disturbance of adaptive functions, virtual life orientation, withdrawal, and tolerance. The SPAI had four subscales, compulsive behavior, functional impairment, withdrawal, and tolerance. The PSUQ had five subscales, dependence, impaired control, denial, decreased productivity, and emotional attachment (Basu et al., 2021). The PMPUQ had four subscales, prohibited use, dangerous use, dependence, and financial problems (Billieux et al., 2008).

Generally, the logic for all the PMPU scales above is that the more symptoms an individual has, the higher the PMPU level he is at. The level of PMPU was indicated by the sum of scores of a scale. Besides, there was some overlap in existing instruments. For example, there are two same dimensions in the MPATS, SAS-C, and SAS-CA, namely, withdrawal and prominent behaviors. Both the SAPS and the SPAI have withdrawal and tolerance dimensions. Moreover, no matter what dimensions a PMPU scale has, the key purpose is to accurately assess the level of PMPU.

In addition, all existing PMPU instruments are traditional paper-and-pencil (P and P) tests based on Classical Test Theory (CTT). In this case, a large number of items are necessary to ensure the accuracy of assessment, and participants should answer all items. Consequently, excessive items would increase the cognitive burden of participants. Also, a long test would lead to boredom and reduce the validity of a test (Lin et al., 2014). Besides, CTT has limitations, such as the estimation of ability being dependent on properties of items and the estimation of item parameters being dependent on participants. Accordingly, two sets of measurement results based on different participants using the same instrument were not comparable (Sun and Guan, 2009). In short, the traditional test is not the optimal way to assess individuals when the instrument is too long and participants are not homogeneous.

To sum up, the existing PMPU measures were all traditional paper-and-pencil tests, which were not convenient to conduct as a large-scale investigation. These PMPU measures were developed in different contexts, although most PMPU measures illustrated a series of same or similar symptoms and shared a common purpose, which was to assess individuals’ PMPU levels.

With the development of Item Response Theory (IRT), computerized adaptive testing (CAT) could provide an optimal solution to psychological assessments with long-scale and heterogeneous samples. Embretson (1996) suggested that CAT was more suitable for measurement of various types of psychological assessment. CAT mainly emphasized that the test was performed by individuals, matched items perfectly with individuals’ trait levels, so it would get the most effective assessment results with a smaller number of items (Weiss, 1982). Many CATs for non-cognitive tests or typical performance tests (e.g., personality tests, mental health tests, emotion regulation tests, subjective well-being tests, self-esteem tests, and social responsibility tests) were already developed (Forbey and Ben-Porath, 2007; Fritts and Marszalek, 2010; Nieto et al., 2018; Wu et al., 2019; Zheng and Cai, 2019; Dai et al., 2020; Xu et al., 2020). Herwin et al. (2022) designed a mobile CAT assessment tool and found that students’ learning motivation could be significantly improved after using the mobile assessment tool, and Miyasawa and Ueno (2013), based on the CAT combined with TTP, designed a mobile testing tool for authentic assessment on the basis of saving time and effort to improve measurement accuracy.

At present, the number of mobile phone users is enormous around the world. Considering the adverse effects of PMPU on mental health, a large-scale instrument for PMPU assessment is necessarily needed (Fumero et al., 2018). Developing a CAT-PMPU (computerized adaptive testing for problematic mobile phone use) tool can provide great convenience for accurate and rapid assessment of PMPU.

The purpose of this study was to develop a CAT-PMPU tool with good psychometric properties. In the first step of this study, a total of 98 items of seven scales were used to develop a PMPU item bank based on IRT. Unidimensionality tests, item calibrations, and differential item functioning analyses were conducted. In the second step, empirical data were used to compare the traditional P and P PMPU tests and the new CAT-PMPU.

## Materials and Methods

### Participants

We recruited 980 participants to complete online or paper-pencil questionnaires. The participants were college students and graduate students from Tianjin, China. Convenience sampling was conducted. The measurement invariance was supported across the paper-pencil questionnaire and the online questionnaire by multiple-group confirmatory factor analysis, so two data sets were analyzed as one. Ninety-five participants were excluded because of not answering more than one item. Finally, a total of 885 valid data were retained, including 318 men and 567 women, and their age ranged from 19 to 25 (*M* = 20.67, *SD* = 2.64).

### Instruments

All scales that are published on journals that were indexed in the Chinese Social Sciences Citation Index (CSSCI), Bei Da He Xin, and Chinese Science Citation Database (CSCD) from the CNKI database in recent 3 years were taken into consideration. The short versions of scales were excluded, since their items were from original versions. Translated versions of the scales that were used in applied studies were excluded, because the development or refinement procedures were not clear and the quality of scale could not be ensured. Finally, seven Chinese version scales of PMPU were included in this study.

#### Chinese Version of the Nomophobia Questionnaire

The Chinese version of the Nomophobia Questionnaire based on the original Nomophobia Questionnaire of Yildirim and Correia (2015) and Ren et al. (2020) revised the Chinese version of Nomophobia Questionnaire (NMP-C) using an exploratory structure equation model (ESEM) and a polytomous item response model. The NMP-C contained 16 items and four dimensions, fear of being unable to access information, losing convenience, losing contact, and losing Internet connection. The NMP-C used a 7-point Likert scale, ranging from 1 (“Not meet at all”) to 7 (“Completely in conformity with”) and had good reliability and validity. Cronbach’s α for the whole scale was 0.948, and for the four dimensions it ranged from 0.867 to 0.916 (Ren et al., 2020). In this study, Cronbach’s α for the whole scale was 0.954.

#### Smartphone Addiction Proneness Scale

The Smartphone Addiction Proneness Scale was a 4-point Likert scale, ranging from 1 (“Strongly disagree”) to 4 (“Strongly agree”), and contained 10 items and four dimensions, disturbance of adaptive functions, virtual life orientation, withdrawal, and tolerance. It had good reliability and validity in empirical studies (Kim et al., 2014). Cronbach’s α for the whole scale was 0.884, and construct validity for the whole scale was great (CFI = 0.962, TLI = 0.902, NFI = 0.943, RMSEA = 0.034). The correlation result between the SAPS and the Mental Health Problems Scale showed that the SAPS has good criterion validity. In this study, Cronbach’s α for the whole scale was 0.838.

#### Smartphone Addiction Inventory

The Smartphone Addiction Inventory was a 4-point Likert scale, ranging from 1 (“Strongly disagree”) to 4 (“Strongly agree”), and contained 20 items and four dimensions, compulsive behavior, functional impairment, withdrawal, and tolerance. Lin et al. (2014) demonstrated it had good reliability and validity. Cronbach’s α for the whole scale was 0.94, and for the four dimensions it ranged from 0.72 to 0.88. The 2-week test–retest reliability of the four subscales ranged from 0.8 to 0.91, and the correlation results among the four subscales of SPAI and the phantom vibration ranged from 0.56 to 0.78, which meant that there was a moderate/high inter-factor correlation (Lin et al., 2014). In this study, Cronbach’s α for the whole scale was 0.926.

#### Mobile Phone Addiction Scale

The Mobile Phone Addiction Scale was a 5-point Likert scale, ranging from 1 (“Never”) to 5 (“Always”), and contained 11 items and four dimensions, inability to control craving, feeling anxious and lost, withdrawal and escape, and productivity loss. It had good reliability and validity. Cronbach’s α for the whole scale was 0.9, and the MPAS was correlated mostly in a hypothesized manner with measures of psychologically meaningful constructs such as leisure, boredom, and sensation-seeking (Leung, 2008). In this study, Cronbach’s α for the whole scale was 0.877.

#### Mobile Phone Addiction Tendency Scale

The Mobile Phone Addiction Tendency Scale was developed by Xiong et al. (2012). It was a 5-point Likert scale, ranging from 1 (“Very inconsistent”) to 5 (“Very well suited to”). It was composed of 16 items and four factors, withdrawal symptoms, salience, social comfort, and mood changes. Previous studies found this scale reliable and valid. Cronbach’s α of the whole scale was 0.83, and for the four dimensions it ranged from 0.81 to 0.92. The result of the confirmatory factor analysis showed that the four-factor model had good fitting indices (CFI = 0.96, RMSEA = 0.07, NFI = 0.94, IFI = 0.96, RFI = 0.93). In addition, researchers have used the MPATS in other studies and proved its high construct validity (CFI = 0.92, TLI = 0.94, RMSEA = 0.07, IFI = 0.96) (Jiang and Bai, 2014). In this study, Cronbach’s α for the whole scale was 0.911.

#### Smartphone Addiction Scale for College Students

The Smartphone Addiction Scale for College Students was a 5-point Likert scale, ranging from 1 (“Strongly unacceptable”) to 5 (“Strongly acceptable”), and contained 11 items and four dimensions, withdrawal behavior, salience behavior, social comfort, and negative effects. Cronbach’ s α for the whole scale was 0.93, and for each factor it ranged from 0.5 to 0.85; the test–rest coefficients were 0.93 for the whole scale and 0.72–0.82 for the four factors. It had great construct validity (CFI = 0.92, TLI = 0.9, RMSEA = 0.05, SRMR < 0.001) (Su et al., 2014). In this study, Cronbach’s α for the whole scale was 0.902.

#### Smartphone Addiction Scale for Chinese Adults

The Smartphone Addiction Scale for Chinese Adults was a 5-point Likert scale, ranging from 1 (“Strongly unacceptable”) to 5 (“Strongly acceptable”), and contained 14 items and four dimensions, withdrawal, salience, social impairment, and somatic discomfort. Cronbach’ s α for the whole scale was 0.909, and for each factor it ranged from 0.714 to 0.814; the test-rest coefficients were 0.931 for the whole scale and 0.743–0.85 for the four factors. It had satisfying structure validity (CFI = 0.94, RMSEA = 0.043, SRMR < 0.001) (Chen et al., 2017). In this study, Cronbach’s α for the whole scale was 0.929.

### Item Response Theory Models

All the PMPU scales in this study were polytomous. Some frequently employed multi-index IRT models for polytomous data involve the graded response model (GRM; Samejima, 1969) and the generalized partial credit model (GPCM; Muraki, 1992). Their item response functions were described as follows.


$$\text{GRM}:P\left(X_{\text{ij}}=\text{t}\right)=P\left(X_{\text{ij}}\geq \text{t}\right)-P\left(X_{\text{ij}}\geq t+1\right),$$

where *P*(*X*_ij_≥*t*) referred to the probability that the subject i will score t or more on item j.


$$P\left(X_{\text{ij}}\geq t\right)=\frac{1}{1+\text{exp}\,\left[-D\,a_{\text{j}}\,\left(\theta_{\text{i}}-b_{\text{jt}}\right)\right]}$$

where α_j_ was the discrimination parameter of the j-th item, bjt was the difficulty parameter of the subject getting t points on item j, and θ*i* was the ability of the subject i.


$$\text{GPCM}:P\left(X_{\text{ij}}=t\right)=\frac{\text{exp}\,\sum_{v=0}^{t}a_{\text{j}}\,\left(\theta_{\text{i}}-\delta_{\text{jv}}\right)}{\sum_{\text{h}=0}^{\text{mj}}\exp\,\sum_{v=0}^{h}a_{\text{j}}\,\left(\theta_{\text{i}}-\delta_{\text{jv}}\right)}$$

where P(X_ij_ = t) represented the probability of subject i to score t on item j, mj was the sum score of item j, t was the subject’s score on this item, δ_jv_ was intersections of two curves of P(X_ij_ = t) and P(X_ij_ = t−1), and it was the only intersection. It may fall anywhere in the θ scale. α_j_ is the discrimination parameter of item j.

### Indices

#### Item Information

Item information is a common concept in IRT. It refers to the information that an item could provide for evaluating participants. More information indicates higher reliability of measurement. In this study, the item selection strategy was the maximum information (MI) method, one of the most widely used in most research studies. The specific content was to select the item with largest information for subjects to answer.

Measurement standard error was inversely proportional to the square of test information, and the formula was as follows:


$$S\,E\,\left(\theta \right)=\frac{1}{\sqrt{\sum_{\text{i}=1}^{\text{m}}{\text{I}}_{\text{i}}\,\left(\theta \right)}}$$

where θ was the level of psychological traits. In this study, subjects’ ability was estimated with the expected *a posteriori* (EAP) method, m was the total number of items, and I_i_(θ) was the information provided by item i to subject with psychological trait level θ.

Some researchers have proposed marginal reliability (MR) based on the test information in IRT, which was easy to use and dynamically monitored the reliability of CAT (Green et al., 1984). The formula is as follows:


$$S\,E=\frac{\sum_{\text{i}=1}^{N}S\,E\,\left(\theta_{\text{i}}\right)}{N}$$

$$M\,R=1-S\,E^{2}$$

where N was the total number of subjects, i was the specific subjects, and SE(θi) was the measurement standard error of subject i at the final estimated θ.

The MR in IRT was the overall reliability of the test calculated by the average measurement error for all subjects rather than CTT’s reliability based on the original score of the test.

#### Stop Rules

The CAT had two stop rules: fixed length and variable length (fixed measurement accuracy) stop rules. In this study, the stop rule of fixed measurement accuracy was adopted, and a total of seven measurement accuracies (*SE* = 0.2/0.3/0.4/0.5/0.6/0.7/0.8) were set. We also designed a stop rule called “all”; that is, participants would terminate the CAT after completing all items in the item bank. Results under the “all” stop rule could be compared with the results under seven stop rules, and then we verified the measurement effectiveness of CAT. To prove the effectiveness and advantage of CAT-PMPU, we also calculated the measurement error of seven traditional P and P PMPU tests and then took them as the stop rule for CAT-PMPU (*SE* = 0.34/0.39/0.55/0.41/0.43/0.36/0.38).

#### Software

SPSS24.0 was used for data preprocessing, principal component analysis (PCA), and unidimensionality test in this study. The R *mirt* package was used to fit the IRT model and calibrate items, the *lordif* package was used to test differential item functioning, and the *catR* package was used to conduct simulation.

## Results

### Unidimensionality Test

**FIGURE 1:**
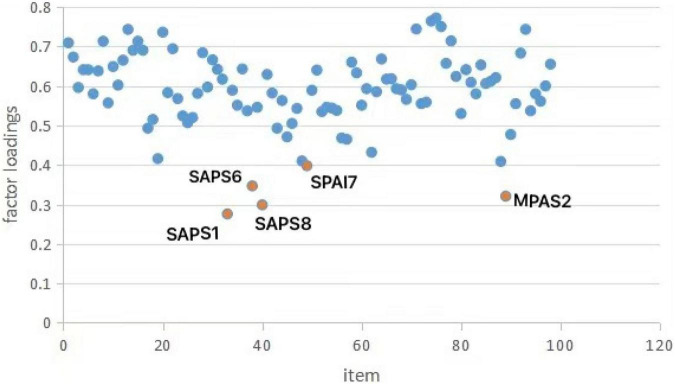
Factor loading of all items.

We deleted some items with poor relevant to problematic mobile phone use in order to ensure the quality of the item bank. First, the loading of each item on the first principal component was obtained by PCA, and items whose loading was lower than 0.4 on first principal component was deleted. Therefore, five items were deleted at this step, three of them were from the SAPS, and the other items were from the SPAI and the MPAS. For example, the content of the MPAS_2 was “I received a mobile bill that I couldn’t afford.” This item was not related to problematic mobile phone use. The factor loading of all items is shown in Figure 1.

Item Response Theory models assumed that the measurement characteristics were unidimensional (Hambleton et al., 1991), so we conducted a unidimensionality test on 93 items. Previous studies indicated that the data could be considered to meet unidimensionality if the ratio of first and second eigenvalues was more than 4 and the variance ratio of the first factor was more than 20% in exploratory factor analysis (EFA) (Reckase, 1979; Andrich, 1996; Reeve et al., 2007). Therefore, we conducted an EFA on the 93 items, and the Kaiser–Meyer–Ollkin (KMO) test result was 0.977, which meant that the 93 items were suitable for factor analysis. Then, the results of the EFA showed that the factor loading of all items was greater than 0.4, the first eigenvalue was 35.631, the second eigenvalue was 6.107, the ratio of the first and second eigenvalues was 5.83, and variance of the first factor accounted for 38.312%, suggesting that the 93 items met unidimensionality.

### Item Response Theory Model Selection

This step compared the model fitting according to fitting indicators such as AIC (Akaike, 1974), BIC (Schwarz, 1978), choosing a model that was relatively more fitting to the data between GPCM and GRM. The smaller the AIC and BIC, the better the model fit.

The specific results are shown in Table 1. Of the two models, the GRM fitted the remaining items best, as it had smaller AIC and BIC values. So GRM was employed to analyze the final CAT-PMPU item bank.

**TABLE 1:** Model fitting index value.

Model	*–2Log-Likelihood*	*AIC*	*BIC*
GPCM	194227.68	195169.7	197423.7
GRM	192627.34	193569.3	195823.4

### Differential Item Functioning Analysis

In order to ensure the quality of items in our item bank, item discrimination analysis and differential item functioning (DIF) analysis were carried out in our study.

Item discrimination referred to the extent in which the item differentiates the actual level of subjects. In our study, items with discrimination less than 0.8 were deleted (Fliege et al., 2005). Four items were removed from item bank, namely, MPATS_1, MPATS_3, SPAI_6, and MPAS_1. The detailed results are shown in Table 2.

**TABLE 2:** Discrimination of deleted items.

Item	Discrimination
MPATS_1	0.799
MPATS_3	0.727
SPAI_6	0.787
MPAS_1	0.777

Differential item functioning was conducted to determine systematic differences caused by demographic variables such as gender and age (Gaynes et al., 2002). Logistic regression was applied to test DIF. Change in McFadden’s pseudo *R*^2^ was employed to test the effect size, the hypothesis of no DIF was rejected when *R*^2^> 0.02, and such items were excluded (Choi et al., 2011). The results showed that none of items’ *R*^2^ values were higher than 0.02. Therefore, there was no DIF according to gender for 89 items. Finally, the 89 items were retained and reanalyzed. The results showed that these items satisfied unidimensionality with no DIF and great discrimination.

### Quality of Item Bank

**FIGURE 2:**
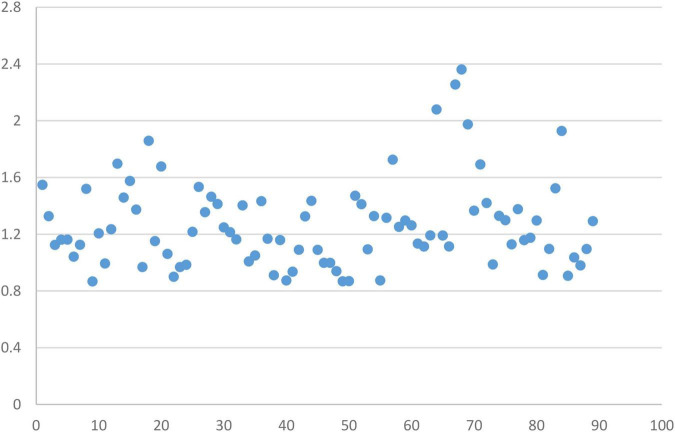
Item discrimination of the item bank.

The discrimination parameters of the remaining 89 items were all > 0.8 with mean of 1.26 (*SD* = 0.31) and from 0.87 to 2.36 with a difference of 1.49, indicating a high-quality item bank (Figure 2). Besides, the difficulty parameters of the items in the item bank were moderate. The specific results of some items are shown in Table 3 below.

**TABLE 3:** Difficulty parameters of some items.

Item	Parameters of difficulty			
	*b1*	*b2*	*b3*	*b4*
SAS_C3	–2.111	–0.191	1.250	3.602
SAS_C6	–2.119	–0.452	0.950	3.444
SAS_C8	–2.206	–0.456	0.954	3.182
SAS_CA4	–2.354	–0.694	0.985	3.450
SAS_CA12	–2.133	–0.442	1.227	3.285
SAS_CA13	–2.119	–0.445	1.150	3.545
MPAS_4	–0.558	0.356	1.715	2.984
MPAS_7	–1.823	–0.849	0.539	2.424
MPAS_8	–1.437	–0.362	1.207	2.986

**FIGURE 3:**
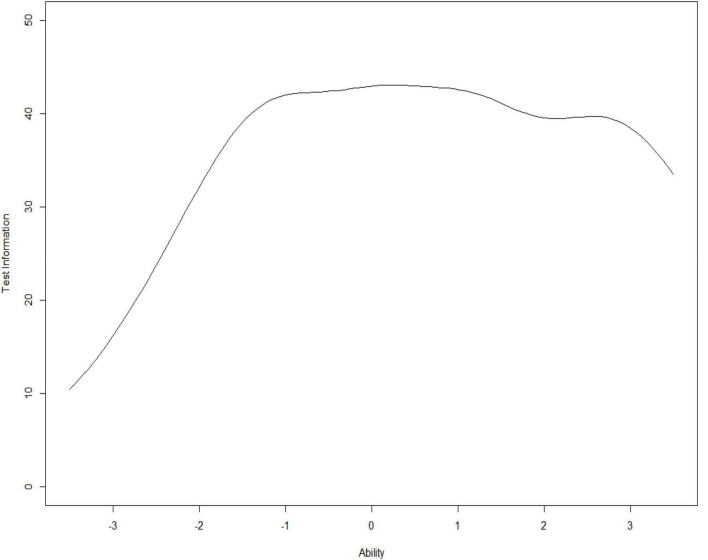
Item bank information curve.

**FIGURE 4:**
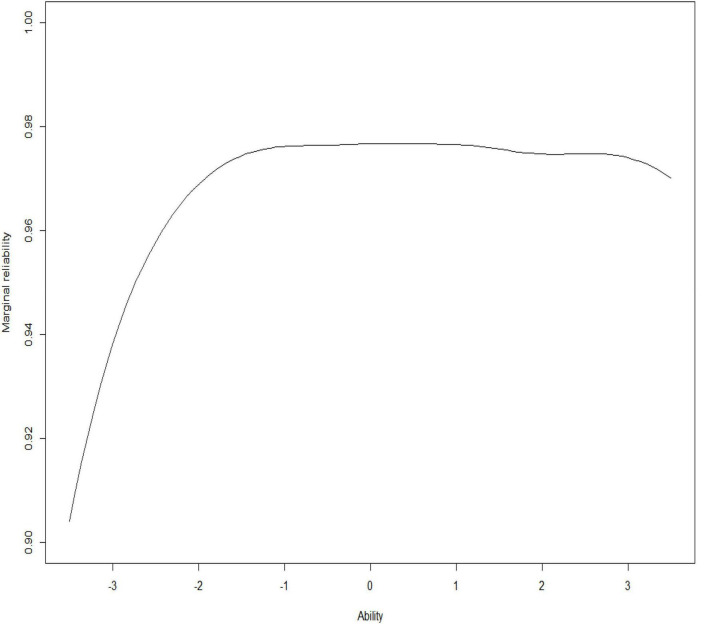
Item bank marginal reliability curve.

This study also calculated the information and marginal reliability of the item bank. The specific results are shown in Figures 3, 4.

It could be seen that the average marginal reliability of the item bank was as high as 0.97, and that the information provided by the item bank was relatively sufficient. It provided high information to subjects whose problematic mobile phone use was at a moderate or upper level and low information to subjects whose problematic mobile phone use was at a low-down level. It showed that the item bank had very high measurement accuracy and reliability for most of the participants. In summary, the quality of the item bank was high and ideal.

### Evaluating Computerized Adaptive Test for Problematic Mobile Phone Use Under Different Stop Rule Conditions

In this study, the specific steps of CAT simulation were as follows: we used the real data obtained by the participants in the P and P test to simulate the CAT; we used the maximum information (MI) as item selection strategy and expected *a posteriori* (EAP) as participants’ ability estimation method, and seven stop rules (*SE* = 0.2/0.3/0.4/0.5/0.6/0.7/0.8) were set.

Table 4 shows the simulation results of real data under several stop rules. It could be seen that under the stop rule of *SE* = 0.5, the correlation coefficient between the ability estimated using 4.07 items and the ability estimated using the entire item bank was as high as 0.93 (*n* = 885, *p* < 0.01), but its marginal reliability was only 0.77, which was not ideal. Under the stop rule of *SE* = 0.4, the correlation coefficient between the ability estimated using 6.93 items and the ability estimated using the entire item bank was as high as 0.95 (*n* = 885, *p* < 0.001), and the measurement reliability was as high as 0.85, so it was recommended to use this stop rule for real CAT. If higher marginal reliability of CAT-PMPU is wanted, the stop rule of *SE* = 0.3 can be used. The correlation coefficient between the ability estimated using 14.27 items and the ability estimated using the entire item bank was as high as 0.97 (*n* = 885, *p* < 0.001), and the marginal reliability reached 0.91, but the number of items used would slightly increase.

**TABLE 4:** Simulation results under seven stop rule conditions.

Stop rule	Number of items used		*Mean SE*	MR	Correlation
	*Mean*	*SD*			
All	89	0.00	0.12	0.99	1.00
*SE*(θ) = 0.2	44.91	7.94	0.20	0.96	0.99
*SE*(θ) = 0.3	14.27	1.91	0.30	0.91	0.97
*SE*(θ) = 0.4	6.93	0.96	0.39	0.85	0.95
*SE*(θ) = 0.5	4.07	0.63	0.48	0.77	0.93
*SE*(θ) = 0.6	2.43	0.56	0.58	0.68	0.88
*SE*(θ) = 0.7	2.05	0.22	0.61	0.63	0.85
*SE*(θ) = 0.8	1.04	0.20	0.74	0.45	0.75

### Comparison of Computerized Adaptive Test for Problematic Mobile Phone Use and Paper-and-Pencil Problematic Mobile Phone Use Tests

This part mainly explored how the accuracy and reliability of CAT-PMPU measurements were improved compared with P and P tests. Measurement accuracy and reliability were compared when the test length of CAT-PMPU was fixed to the same length as the traditional P and P PMPU test. For example, NMP contained 16 items; the measurement error and marginal reliability could be calculated when NMP was used alone to estimate subject’s ability. At the same time, a fixed-length stop rule (16 items) could be used in CAT-PMPU, and the corresponding measurement error and marginal reliability could be calculated. Then, the measurement accuracy and reliability of traditional P and P PMPU test improved by CAT-PMPU could be compared under the same test length.

#### Measurement Error of Two Test Methods Under Same Test Length

The measurement error results of the traditional P and P PMPU test and CAT-PMPU with the same test length are shown in Table 5. It could be seen that compared with the P and P PMPU test, CAT-PMPU significantly reduced the measurement error by 16.7–34.1% (*M* = 24.4%) (*p* < 0.001, Cohen’s *d* > 1). The measurement error of the traditional P and P PMPU test was high when test length was short. Measurement accuracy could be greatly improved from the unacceptable value in the original P and P test to an acceptable value by CAT-PMPU. For example, the measurement error of the traditional P and P PMPU test was 0.55 in 7-item SAPS, which was poor and unacceptable, while the measurement error of CAT-PMPU was 0.39.

**TABLE 5:** Measurement error of two test methods under same test length.

Method	Length	Measurement error			
		Mean (*SD*)	*SE* decrease	*t*	Cohen’s *d*
NMP	16	0.34 (0.039)	17.6%	44.54***	1.50
CAT-PMPU	16	0.28 (0.014)			
MPATS	14	0.39 (0.036)	23.1%	79.49***	2.67
CAT-PMPU	14	0.30 (0.015)			
SAPS	7	0.55 (0.027)	29.1%	141.07***	4.74
CAT-PMPU	7	0.39 (0.023)			
SPAI	18	0.41 (0.025)	34.1%	150.12***	5.05
CAT-PMPU	18	0.27 (0.013)			
SAS-C	11	0.43 (0.041)	23.3%	69.19***	2.33
CAT-PMPU	11	0.33 (0.017)			
SAS-CA	14	0.36 (0.041)	16.7%	45.99***	1.55
CAT-PMPU	14	0.30 (0.015)			
MPAS	9	0.48 (0.043)	27.1%	85.58***	2.88
CAT-PMPU	9	0.35 (0.020)			

***p < 0.001.

#### Measurement Reliability of Two Test Methods Under Same Test Length

The measurement reliability results of the traditional P and P PMPU test and CAT-PMPU with the same test length are shown in Table 6. It could be seen that compared with the P and P PMPU test, CAT-PMPU significantly improved the measurement reliability by 4.5–23.2% (*M* = 11%) (*p* < 0.001, Cohen’s *d* > 1). The measurement reliability of the P and P PMPU test was low when test length was short. Measurement reliability could be greatly improved from the unacceptable value in the original P and P PMPU test to an acceptable value by CAT-PMPU. For example, the measurement reliability of the P and P PMPU test was 0.69 in seven-item SAPS, which was poor and unacceptable, while the measurement reliability of CAT-PMPU was 0.85.

**TABLE 6:** Measurement reliability of two test methods under same test length.

Method	Length	Measurement reliability			
		Mean (*SD*)	Reliability increase	*t*	Cohen’s *d*
NMP	16	0.88 (0.028)	4.5%	40.00***	1.34
CAT-PMPU	16	0.92 (0.008)			
MPATS	14	0.84 (0.029)	8.3%	69.54***	2.34
CAT-PMPU	14	0.91 (0.009)			
SAPS	7	0.69 (0.031)	23.2%	131.60***	4.42
CAT-PMPU	7	0.85 (0.019)			
SPAI	18	0.83 (0.022)	12.0%	124.27***	4.18
CAT-PMPU	18	0.93 (0.007)			
SAS-C	11	0.82 (0.035)	9.8%	63.43***	2.13
CAT-PMPU	11	0.90 (0.011)			
SAS-CA	14	0.87 (0.031)	4.6%	41.10***	1.38
CAT-PMPU	14	0.91 (0.009)			
MPAS	9	0.77 (0.043)	14.3%	75.05***	2.52
CAT-PMPU	9	0.88 (0.014)			

***p < 0.001.

The results showed that CAT-PMPU could greatly reduce measurement error and improve measurement accuracy under the same test length; that was to say, CAT-PMPU could achieve higher measurement accuracy than the P and P test if we use the scale with same length.

#### Comparison of the Accuracy of Computerized Adaptive Test for Problematic Mobile Phone Use and Each Paper-and-Pencil Problematic Mobile Phone Use Test

The ability estimation values were used to evaluate the accuracy of CAT-PMPU and P and P tests. First, the estimation values were calculated from original PMPU tests. Second, the length of the original PMPU tests was used as stop rule for CAT-PMPU. Then, seven sets of ability estimation values of seven CAT-PMPU tests could be gained. Eventually, the means and standard deviations from the seven P and P tests and seven CAT-PMPU could be compared. The correlations between each P and P test and CAT-PMPU were calculated. Besides, the ability estimation results based on all the 89 items were presented as a baseline. The results from Table 7 show that the correlations between CAP-PMPU and the P and P tests ranged from 0.78 to 0.92. The means of ability estimation from all the tests were approximate, around −0.2. It was noteworthy that the ability estimation results from seven CAT-PMPU were even better than those of the seven original P and P tests when compared with the 89-item test.

**TABLE 7:** Ability means, SDs, and correlation coefficients of computerized adaptive testing (CAT) and paper-and-pencil (P and P) tests under the same test length.

		P&P PMPU								CAT PMPU						
		All	NMP	MPATS	SAPS	SPAI	SASC	SASCA	MPAS	16-item test	14-item test	7-item test	18-item test	11-item test	14-item test	9-item test
	Mean	–0.24	–0.19	–0.20	–0.17	–0.22	–0.20	–0.21	–0.16	–0.21	–0.21	–0.18	–0.24	–0.21	–0.20	–0.20
	*SD*	1.35	1.68	1.47	1.29	1.42	1.48	1.69	1.35	1.22	1.24	1.19	1.26	1.22	1.25	1.21
P&P PMPU	NMP	0.83														
	MPATS	0.92	0.74													
	SAPS	0.82	0.63	0.77												
	SPAI	0.86	0.58	0.77	0.76											
	SASC	0.88	0.63	0.82	0.69	0.81										
	SASCA	0.93	0.74	0.87	0.74	0.81	0.87									
	MPAS	0.83	0.76	0.77	0.66	0.65	0.71	0.78								
CAT PMPU	16-item test	0.98	0.82	0.90	0.79	0.84	0.86	0.92	0.82							
	14-item test	0.97	0.82	0.89	0.81	0.84	0.86	0.91	0.81	0.95						
	7-item test	0.95	0.80	0.88	0.78	0.82	0.84	0.89	0.79	0.93	0.93					
	18-item test	0.98	0.83	0.90	0.80	0.84	0.86	0.92	0.82	0.96	0.95	0.94				
	11-item test	0.96	0.81	0.89	0.78	0.82	0.85	0.91	0.80	0.94	0.94	0.92	0.94			
	14-item test	0.97	0.82	0.90	0.79	0.84	0.86	0.91	0.81	0.95	0.95	0.93	0.96	0.95		
	9-item test	0.96	0.81	0.89	0.79	0.82	0.85	0.91	0.81	0.94	0.94	0.92	0.95	0.93	0.94	

#### Comparison of the Effectiveness of Computerized Adaptive Test for Problematic Mobile Phone Use and Each Paper-and-Pencil Problematic Mobile Phone Use Test

In order to compare the effectiveness between CAT-PMPU and each P and P test, the item usage of two test methods was calculated. Specifically, measurement errors of the seven P and P tests were calculated as stop rules for CAT-PMPU. The average item usage for CAT-PMPU under the seven stop rules was calculated. The specific results were shown in Table 8, take P and P SPAI for instance, when individuals finished all 18 items, the accuracy of PMPU was 0.41 (*SE* = 0.41). By comparison, individuals only needed to complete 6.54 items to reach the same accuracy as P and P SPAI.

**TABLE 8:** Simulation results under seven stop rules based on P and P problematic mobile phone use (PMPU) tests.

Accuracy	P&P PMPU length	CAT-PMPU length
*SE*(NMP) = 0.34	16.00	10.37
*SE*(MPATS) = 0.39	14.00	7.35
*SE*(SAPS) = 0.55	7.00	3.25
*SE*(SPAI) = 0.41	18.00	6.54
*SE*(SASC) = 0.43	11.00	5.89
*SE*(SASCA) = 0.36	14.00	8.96
*SE*(MPAS) = 0.48	9.00	4.58

## Discussion

This study developed a computerized adaptive testing tool for problematic mobile phone use based on IRT. A simulation study based on an empirical dataset showed that the length of CAT-PMPU was much shorter than that of a P and P test, and that measurement errors were also much lower. Compared with the length of the seven P and P PMPU scales, CAT-PMPU reduced the test length, which further proved the advantages and effectiveness of CAT-PMPU. The CAT-PMPU developed in this study could reduce the cognitive burden of participants by reducing item consumption without decreasing the accuracy of PMPU assessment. That is an advantage of CAT (Pilkonis et al., 2014).

In this study, the participants only needed to complete four items in CAT-PMPU, and the estimated ability level was as high as 0.93 compared with the estimated ability level of completing all 89 items in the traditional P and P test. These results were similar to the results of CAT in other fields. For example, in computerized adaptive testing for college student social responsibility, participants only needed to answer 5.84 items to be highly correlated with the estimated ability of all items (Dai et al., 2020). It can be seen that CAT-PMPU will be easily used for large-scale investigations.

Compared with traditional P and P tests, CAT-PMPU offered different items for participants when measurement standard error was fixed at an acceptable level (for example, 0.3 and 0.4). Specifically, the individuals’ PMPU estimated from the new CAT-PMPU was significantly correlated with those estimated from a traditional full PMPU test (*SE* < 0.5, *r* = 0.93). It indicated that in CAT-PMPU, individuals’ PMPU levels could be accurately assessed with much less items with higher discrimination. In fact, if a little higher measurement error was permitted, less items would be used (92.2% of items were reduced when *SE* < 0.4, and 95.4% of items were reduced when *SE* < 0.5). This result was similar to some previous studies, i.e., in the computerized adaptive testing for self-esteem, the individuals’ ability estimated based on 9 items was significantly correlated with the ability estimated based on a full test (*SE* < 0.45, *r* = 0.91; *SE* < 0.32, *r* = 0.94) (Zheng and Cai, 2019).

There were some shortcomings needed to be further discussed. In this study, participants’ PMPU levels were estimated with fixed item parameters. Ignorance of estimation errors may lead to lower measurement accuracy. Niu and Choi (2022) proposed that performing the fully Bayesian adaptive testing with a revised proposal distribution would greatly reduce measurement errors and attain better estimation accuracy at the lower end of the ability scale. To get better measurement accuracy, this method will be employed in the future study.

The item selection strategy used in this study was MI, which was used by a large body of researchers (Luo et al., 2019; Zhang et al., 2020). However, this strategy prefer high discrimination items, which might result in overexposure of some items. The shadow-test approach or freezing shadow test approach that added random item-ineligibility constraints to the model would be a good solution to item exposure (Choi et al., 2021; van der Linden, 2021). Although PMPU is not a high-risk test, item exposure control techniques will be necessary when sample size keeps increasing.

Besides, PMPU tests are symptom-based tests. An individual always presents several symptoms at the same time. That means, items in PMPU tests are not thoroughly stand-alone. How to deal with this situation is a challenge for psychometricians (Choi and Lim, 2021). With augmentation of the item bank in the future, CAT-PMPU with adaptive test assembly methods will be taken into consideration.

## Conclusion

The CAT-PMPU developed in this research meets the requirements of IRT measurement characteristics, and its measurement accuracy and efficiency are significantly better than those of P and P tests for problematic mobile phone use. In addition, CAT-PMPU can significantly improve measurement accuracy, which provides a new technical support for actual measurement of problematic mobile phone use.

## Data Availability Statement

The original contributions presented in the study are included in the article/Supplementary Material, further inquiries can be directed to the corresponding author.

## Ethics Statement

The studies involving human participants were reviewed and approved by Ethics Committee of Tianjin University (XL2020-12). The patients/participants provided their written informed consent to participate in this study.

## Author Contributions

XL and TL contributed to conception and design of the study. ZZ organized the database. XL performed the statistical analysis and wrote the first draft of the manuscript. TL, HL, and MC revised the first draft of the manuscript. All authors contributed to manuscript read, and approved the submitted version.

## Conflict of Interest

The authors declare that the research was conducted in the absence of any commercial or financial relationships that could be construed as a potential conflict of interest.

## Publisher’s Note

All claims expressed in this article are solely those of the authors and do not necessarily represent those of their affiliated organizations, or those of the publisher, the editors and the reviewers. Any product that may be evaluated in this article, or claim that may be made by its manufacturer, is not guaranteed or endorsed by the publisher.
