# Supplementary material for: Development of a Computerized Adaptive Test for Problematic Mobile Phone Use
Source: Front Psychol. 2022 May 31;13:892387. doi: 10.3389/fpsyg.2022.892387 (PMC9197499; doi:10.3389/fpsyg.2022.892387)
Supplement: Supplementary file 2 [file Table_2.docx]

We are more than seven scale for the multi-group CFA, the table below for the result of NMP scale, according to the model fitting index we can find that, chi-square vulnerable to the influence of sample size, with the increase of sample size even small differences will also get significant results, therefore, we adopt the method of fitting index difference detection measurement equivalence, When the difference is less than 0.01, there is no significant difference, and the variation of Δ DF, ΔTLI, Δ CFI and ΔRMSEA among models in this data analysis is all less than 0.01. Therefore, we can conclude that the data of paper version and network questionnaire version have the measurement equivalence

**Table 1** Equivalence fitting index between NMP paper version and online questionnaire version

| Model | *S-Bχ^2^* | *Df* | *TLI* | *CFI* | *RMSEA (90% CI)* |
| --- | --- | --- | --- | --- | --- |
| A Configural Invariance | 1075,601*** | 196 | .906 | .923 | .100 (.094, .106) |
| B Metric Invariance | 1075,601*** | 196 | .906 | .923 | .100 (.094, .106) |
| C Scalar Invariance | 610.357*** | 220 | .909 | .917 | .098 (.092, .103) |
| Model comparison | *Δχ^2^* | *Δdf* | *ΔTLI* | *ΔCFI* | *ΔRMSEA* |
| A vs B | 0 | 0 | 0 | 0 | 0 |
| B vs C | 465.244 | 24 | .003 | .006 | .002 |
